# Irreducible Solid Electrolytes: New Perspectives on Stabilizing High-Capacity Anodes in Solid-State Batteries

**DOI:** 10.1021/acsenergylett.5c02289

**Published:** 2025-10-10

**Authors:** Wenxuan Zhao, Anastasia K. Lavrinenko, Meng-fu Tu, Lucas Huet, Alexandros Vasileiadis, Theodosios Famprikis, Marnix Wagemaker, Swapna Ganapathy

**Affiliations:** Radiation Science and Technology, Faculty of Applied Sciences, 2860 Delft University of Technology, Delft, 2629 JB, The Netherlands

## Abstract

Irreducible solid electrolytes (SEs), characterized by non-Li framework ions in their lowest oxidation states, offer intrinsic compatibility with low-reduction-potential, high-capacity negative electrodes, such as lithium metal and silicon. In these SE materials, disorder engineering and vacancy formation reduce lithium-ion diffusion barriers, achieving room-temperature ionic conductivities exceeding 0.1 mS cm^–1^. Experiments and atomistic simulations confirm that irreducible SEs form decomposition-free interfaces with Li metal. Their limited oxidative stability can be addressed by pairing them with an electrolyte layer stable with practical cathodes yet demanding interface compatibility between the two electrolyte layers. Here we highlight key research directions to accelerate irreducible SE transition from laboratory to practical application, including expanding compositional diversity, optimizing interfaces with cathode-facing electrolytes, developing scalable thin-film processing, and exploring compatibility with other low working potential anodes like silicon. Addressing these challenges is essential to unlock the full potential of irreducible SEs for high-energy, long-life, all-solid-state batteries.

## Graphical abstract

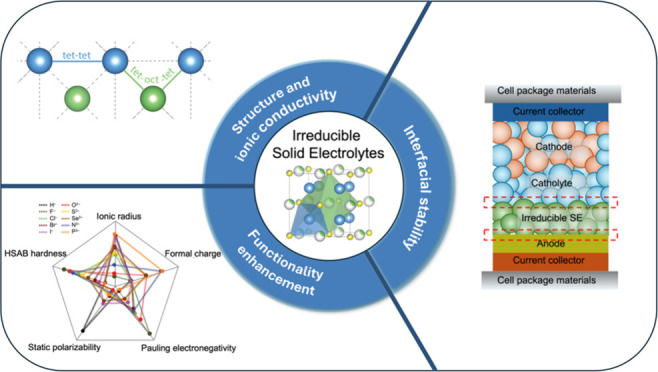

All-solid-state batteries (ASSBs) promise safe, high-energy density storage by pairing high-capacity anodes with nonflammable ceramic solid electrolytes (SEs).^1^ Their gravimetric energy density could exceed 350 Wh kg^–1^ (ref 2), and removal of liquid electrolytes raises the onset temperature of thermal runaway,^3,4^ positioning ASSBs at the forefront of next-generation battery research.

These expectations put high demands on the properties of the SEs, i.e., liquid-like ionic conductivities (≥1 mS cm^–1^) and electrochemical stability across the full-cell voltage. Oxyhalides^5,6^ (e.g., LiTaOCl_4_) and halogen-rich argyrodites^7–10^ (e.g., Li_5.3_PS_4.3_Br_1.7_) already achieve conductivities of ∼10 mS cm^–1^, yet their electrochemical stability window (ESW) is limited, owing to fragile anionic (e.g., S^2–^) or cationic frameworks (e.g., Ta^5+^, P^5+^) upon oxidation or reduction, respectively.^11,12^ While high-potential-tolerant halide SEs^13–15^ and protective coatings^16^ improve electrochemical compatibility at the positive electrode, their development and application at the negative electrode remain challenging.

Li metal and Si-based anodes achieve exceptional capacities but demand (electro)­chemical passivation down to ∼0 V vs Li/Li^+^.^17,18^ However, common SEs, such as sulfides, oxides, and halides, typically reduce to low-valence states or even elemental metals at low potentials.^11^ If the resulting products are mixed ion-electron conductors, reduction can propagate, leading to continuous SE decomposition.^19^ Certain SEs, such as sulfur-based argyrodites,^20^ lithium phosphorus oxynitride (LiPON)^19^ and hydrides,^21^ instead exhibit kinetic stability by forming electronically insulating and self-limiting interphases. For example, Li_6_PS_5_Cl decomposes into poorly electronconductive products such as LiCl, Li_2_S, and Li_3_P,^12^ while LiBH_4_-[LiNBH]_*n*_ systems generate passivation layers of Li–B and Li_3_N^22^ in situ. Although such interphases suppress further decomposition, they inevitably increase interfacial resistance and consume active Li, often necessitating prelithiation strategies to compensate for the associated Li loss.

Comparably, an evident strategy to mitigate anode-side instability is to design SEs that are inherently stable against low-potential anodes. This requirement is fulfilled if all elements of the SE, except for Li-ions, are in their lowest oxidation state and thus irreducible (e.g., the lithium binaries LiX with X = N^3–^, O^2–^, S^2–^, Cl^–^). Recent advances have expanded the landscape of such irreducible SEs, including antifluorite-type materials like Li_3_N-LiX (X = Cl, Br), Li_3_N–Li_2_S, Li_3_P–Li_2_S and antiperovskites^17,23–28^ (Figure 1).

**Figure 1:**
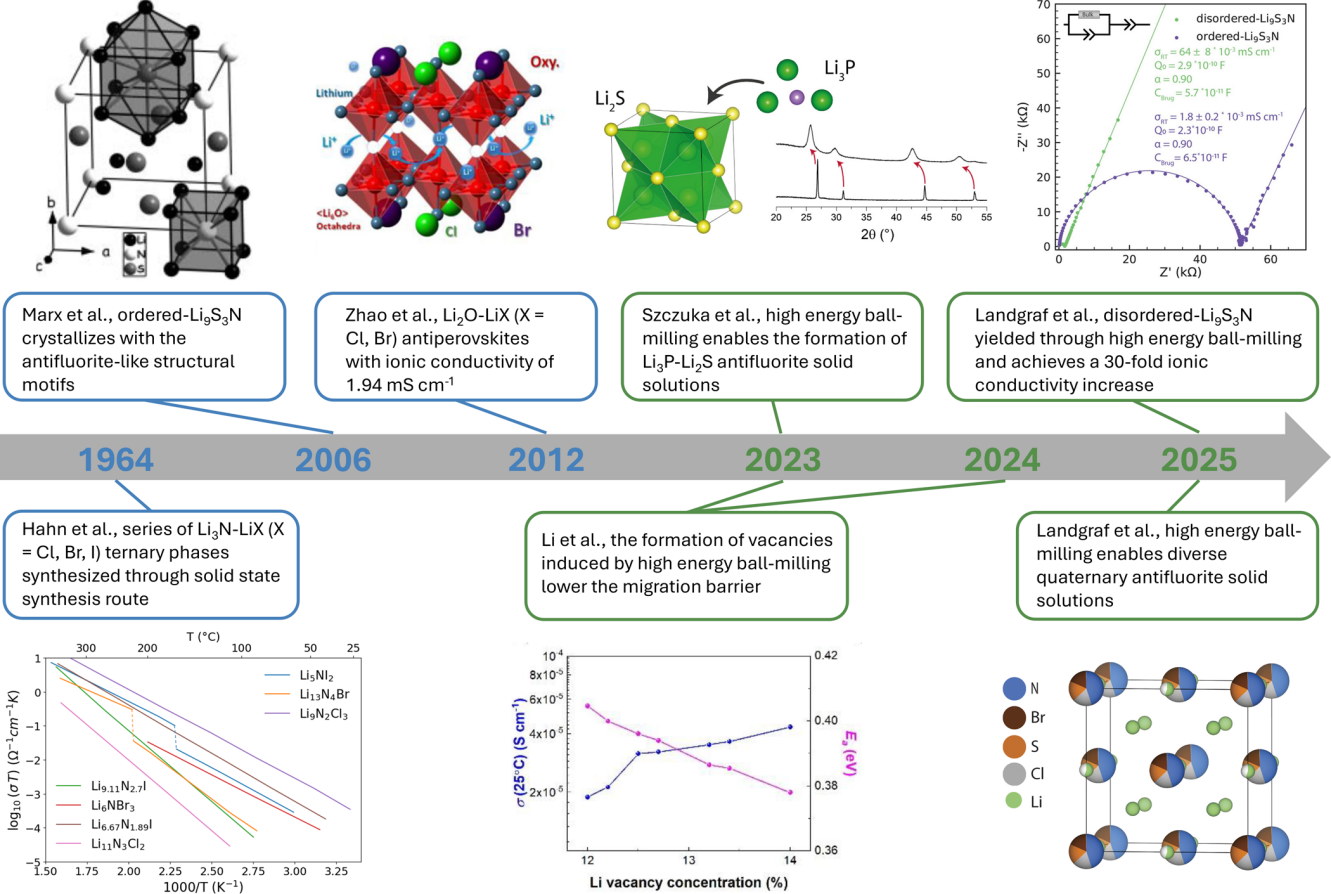
Timeline of the development of irreducible SE families. Adapted with permission from ref 31. Copyright 1981 Elsevier. Reprinted with permission from ref 32. Copyright 2006 Wiley-VCH Verlag GmbH & Co. KGaA. Reprinted with permission from ref 23. Copyright 2012 American Chemical Society. Reprinted with permission from ref 28. Copyright 2023 American Chemical Society. Reprinted with permission from ref 24. Available under a CC BY-NC 4.0. Copyright 2023 American Association for the Advance of Science. Reprinted with permission from ref 29. Copyright 2024 Springer Nature. Reprinted with permission from ref 27. Available under a CC-By 3.0. Copyright 2025 Royal Society of Chemistry. Reprinted with permission from ref 26. Copyright 2025 American Chemical Society.

Mechanochemical synthesis introducing structural and compositional disorder has substantially boosted their room-temperature ionic conductivities.^26–28^ As intended, these materials exhibit excellent electrochemical stability down to ∼0 V vs Li/Li^+^ and show no reactivity or solid electrolyte interface (SEI) formation upon direct contact with Li metal.^17,25,29,30^ Practical cell application, however, also demands oxidative stability during delithiation of the anode (e.g., up to ∼1 V vs Li/Li^+^ for silicon^18^) and interfacial compatibility with the positive electrode. Since the oxidative stability of current irreducible SEs is inherently limited, the solution is paring them with a catholyte (cathode-facing electrolyte) yet introducing new interface compatibility challenges.

In this Perspective, we critically assess recent experimental and computational advancements in irreducible SEs, identify prevailing challenges, and outline key research directions aimed at fully exploiting their potential and enabling their transition from lab materials to practical application in next-generation ASSBs.

The most well-known irreducible compounds are lithium binaries such as lithium halides (e.g., LiCl), chalcogenides (e.g., Li_2_S), and pnictides (e.g., Li_3_N). Since the 1960s, the solid-state synthesis route, in which the reactants are annealed under high temperature, has yielded numerous ternary phases along the Li_3_N-LiX (X = Cl, Br, I) tie line^31^ (Figure 1). Later, similar methods led to the discovery of ternary compounds in the Li_3_N–Li_2_S^32^ and Li_2_O–LiX^23^ systems. However, only select compounds like α-Li_3_N (hexagonal *P*6/*mmm*)/β-Li_3_N (antifluorite),^29^ Li_7_N_2_I,^33^ and certain antiperovskites^23^ exhibit room-temperature ionic conductivities above 0.1 mS cm^–1^, hindering practical application. Moreover, reported conductivities differ by more than an order of magnitude for the latter two compounds, underscoring reproducibility challenges.^34,35^

Among the emerging families of irreducible SEs antifluorite-type phases have recently attracted considerable attention due to their ionic conductivities achieving ∼1.0 mS cm^–1^ and inherent structural and compositional flexibility, which can further enhance lithium-ion diffusion. In addition, many recent studies have investigated ionic transport, the role of disorder, and electrochemical stability within antifluorites,^17,24–27,36^ motivating the focus on this motif as a representative case study.

In the anion-disordered lithium-rich antifluorite structure (Figure 2a), Li^+^ primarily occupies tetrahedral sites (Li-tet), while energetically less favorable octahedral sites (Li-oct) are only partially occupied. Density functional theory (DFT) and *ab initio* molecular dynamics (AIMD) identify two main migration pathways (Figure 2b): (1) direct hopping between tetrahedral sites (tet-tet) and (2) via interstitial octahedral positions (tet-oct-tet).^26,27,30^

**Figure 2:**
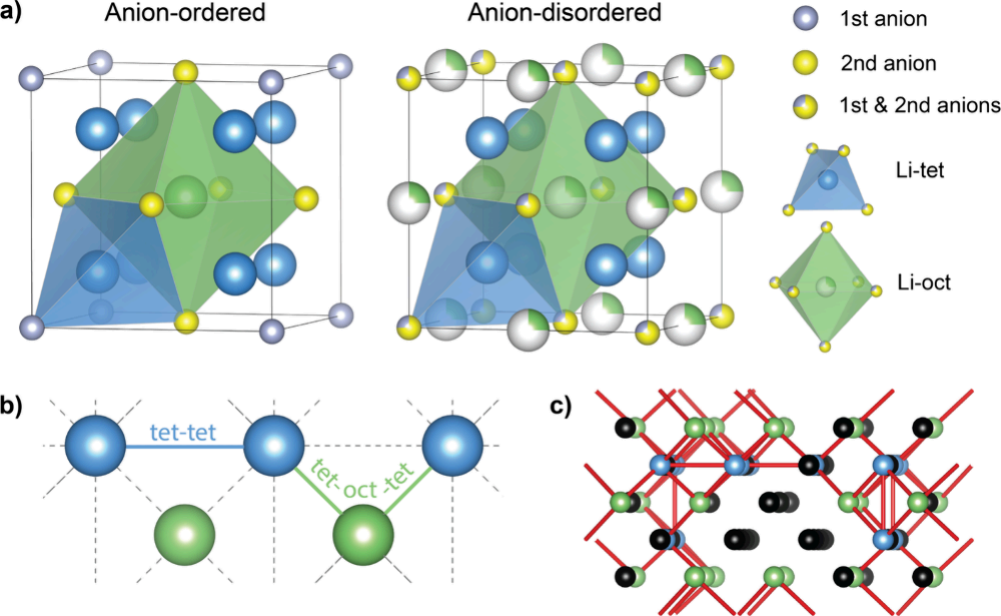
Structure and ion transport in lithium-rich irreducible antifluorite SEs. (a) Ordered and disordered configurations in a ternary compound with two anion species (e.g., S^2–^ and N^3–^). Lithium occupies tetrahedral (blue) and octahedral (green) sites, while anions are shown in yellow and violet. (b) The two primary lithium migration pathways. (c) Example of a percolation network, where active tetrahedral (blue) and octahedral (green) lithium sites form a continuous conduction pathway (connected by red jumps), while isolated Li sites (black) remain disconnected at the chosen energy cutoff.

Compositional and structural disorder can stabilize these high-energy octahedral sites, widen diffusion bottlenecks and create low-energy pathways for long-range diffusion by introducing diversity of local environments. For example, partial substitution of larger anions (e.g., Cl^–^ or S^2–^) with smaller ions (e.g., N^3–^) enlarges diffusion bottlenecks,^26,27^ while introducing larger anions (e.g., Br^–^ instead of Cl^–^ in Li_2.31_S_0.41_Br_0.14_N_0.45_) increases lattice parameters and polarizability,^27^ both relieving steric constraints for ion migration. Substitution with higher oxidation state anions (e.g., S^2–^ with P^3–^) increases lithium content and modifies Coulombic interactions in the system, stabilizing octahedral Li sites and reducing migration energy barriers.^30^ Anion-disordered arrangement (e.g., N^3–^ and S^2–^ sharing the same crystallographic position) diversifies local coordination environments, creating energetically favorable diffusion pathways absent in anion-ordered configurations (Figure 2a). The percolation model further demonstrates that compositional and structural disorder in Li_2+*x*_S_1–*x*_N_*x*_ exhibits a lower percolation-onset energy and a higher fraction of lithium ions connected to the percolation network (Figure 2c illustrates an example of percolation network), thereby enhancing ionic conductivities.^26^

Compositional and structural disorder, typically introduced through mechanochemical synthesis, effectively lowers the Li-ion diffusion energy barrier and, in turn, boost the ionic conductivity of irreducible antifluorite SEs.

To obtain these metastable and highly disordered phases, irreducible antifluorite SEs are typically synthesized through mechanochemistry instead of traditional high-temperature solid-state routes.^26^ Along the LiCl–Li_3_N, Li_2_S–Li_3_N, Li_2_S–Li_3_P, and LiX–Li_2_S–Li_3_N (X = Cl, Br) tie lines, this approach has yielded a diverse range of disordered ternary and quaternary solid solutions with enhanced ionic conductivities (Figure 1). For example, high-energy milling induces structural disorder in Li_2+*x*_S_1–*x*_N_*x*_, resulting in orders of magnitude increase in ionic conductivity from 0.002 to 0.2 mS cm^–1^ (ref 26). In β-Li_3_N^29^ and Li_9_N_2_Cl_3_,^24^ it has been proposed that high-energy ball-milling provides the energy required to create lithium and nitrogen vacancies that lower the diffusion barrier and increase ionic conductivity by an order of magnitude.

The largely unexplored chemical space and high structural flexibility of irreducible antifluorite SEs allow to tune the atomic scale interactions that govern ion transport. As a result, this class of materials provides future opportunities to systematically explore structure-performance relationships and to design novel irreducible SEs for next generation ASSBs.

The high structural flexibility of irreducible antifluorite SEs offer a promising opportunity to simultaneously enhance ionic conductivity and electrochemical stability through rational selection of anions.

Reversible cycling of high-energy-density ASSBs requires stable electrode|SE interfaces across the full cell voltage, thus demanding a SE with a sufficiently wide ESW to cover the working potentials of both electrodes. This prevents undesirable redox reactions, Li inventory loss, and increased impedance induced by less conductive decomposition products.^37^ Li metal presents the most stringent negative electrode active material for reduction stability, featuring the lowest standard reduction potential for Li^+^ (−3.04 V vs standard hydrogen electrode).^17^ Typical oxidation potentials exceed 4 V vs Li/Li^+^ depending on the precise choice of the positive electrode active material.

A rapid and cost-effective approach to estimate ESW of SEs is to employ atomistic simulations.^38^ For irreducible binaries such as Li_3_N and Li_2_S, thermodynamic calculations based on the decomposition products predict a stability down to 0 V vs Li/Li^+^, confirming their intrinsic compatibility with Li metal anodes.^39^ Similarly, the grand-potential phase diagram shows that Li_2.5_N_0.5_S_0.5_ remains thermodynamically stable at 0 V vs Li/Li^+^ (Figure 3a),^17^ unlike commonly studied sulfides, halides, oxides, and LIPON-type SEs which tend to get reduced.^40^ Experimental cyclic-voltammetry (CV) scans of symmetric Li|Li_2+*x*_S_1–*x*_N_*x*_|Li cells further show no reduction peaks down to 0.1 V vs Li/Li^+^, corroborating their electrochemical irreducibility.^26^

**Figure 3:**
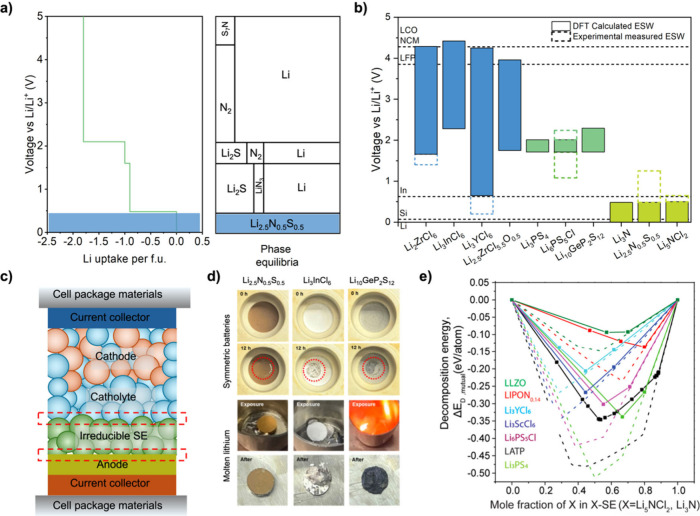
Interfacial stability of irreducible SEs. (a) Grand-potential phase diagram and equilibrium voltage profile of Li_2.5_N_0.5_S_0.5_. The blue part denotes the thermodynamic stability down to 0 V vs Li/Li^+^. Adapted with permission from ref 17. Copyright 2024 American Chemical Society. (b) ESWs for representative SEs, reported in the literature:^11,17,26,27,29,39,41,42^ solid bars indicate first-principles predictions; open bars show voltametric measurements. The dashed lines indicate the typical reduction limit of anodes and oxidation limit of cathodes. (c) Schematic illustration of an anode|irreducible SE|catholyte|cathode stack configuration. (d) Optical images of SE pellets before testing, after 12 h of aging in Li|SE|Li symmetric cells, and after being exposed to molten Li. Reprinted with permission from ref 17. Copyright 2024 American Chemical Society. (e) Reaction energies for pseudobinary mixtures of Li_5_NCl_2_ (solid line) and Li_3_N (dashed line) with several highly conducting SEs. Reprinted with permission from ref 25. Copyright 2023 American Chemical Society.

As summarized in Figure 3b, irreducible SEs consistently exhibit excellent reduction stability, aligning with their chemistry-by-design principle (fully reduced anions). However, their oxidative stability is inherently limited. Phase-stability calculations predict the oxidation limits of ∼0.5 V vs Li/Li^+^ for Li_3_N, Li_3_N–LiCl and Li_3_N–Li_2_S.^17,25,39^ Although experimental linear sweep voltammetry (LSV) measurements revealed extended oxidation stability limits of ∼0.65 V for Li_3_N–LiCl^27^ and ∼2 V for Li_3_N–Li_2_S,^26^ these values are still insufficient for directly pairing with ≥4 V cathodes. Notably, computational ESW estimations, based on the total energy of reactant SE and decomposition products, account only for the thermodynamic driving forces and neglect kinetic barriers, typically underestimating the ESW. By incorporating the kinetic stability into the ESW analysis, wider stability limits are often predicted, showing good agreement with experimental stability data across diverse classes of SEs^38^ and even predicting the limits of reversible (de)­lithiation.^11^ Therefore, further experimental and theoretical studies remain essential to fully understand and optimize the oxidative stability of irreducible SEs.

Given that oxidation-induced degradation remains a challenge at high potentials for current irreducible SEs, a promising engineering solution is to employ a bilayer configuration. Recent studies have demonstrated that bilayer designs can suppress interfacial degradation, broaden the ESW, and enable long-term cycling.^43^ For example, pairing an argyrodite catholyte with a hydride-based anolyte Li_5_PS_4_(BH_4_)_2_ enhances interfacial stability toward Li metal and delivers longer cycling than cells with Li_6_PS_5_Cl alone.^44^ Translating this concept into irreducible SEs, one could envision integrating a more oxidatively robust halide or sulfide catholyte to stabilize the high-potential interface (i.e., an irreducible SE|catholyte bilayer, as illustrated in Figure 3c). In this way, the irreducible SE would only need to remain stable above the catholyte’s reduction potential (e.g., 0.6 V for Li_3_YCl_6_ ^45^ or 1.08 V for Li_6_PS_5_Cl^11^), thereby relaxing the oxidative stability requirement for irreducible SEs while leveraging its intrinsic reduction stability at the anode side.

Although an irreducible SE|catholyte bilayer stack can theoretically secure a desired ESW that spans the full-cell operation voltage, ESW alone does not guarantee interfacial compatibility and cycling durability. The chemical stability of the SE with adjacent components (e.g., anode and pairing catholyte chemistries), as well as cycle performance of the resulting bilayer electrolyte, are equally critical.

Recent studies have investigated the cyclability of irreducible SE layers such as Li_3_N and Li_3_OCl, significantly suppressing interfacial degradation and broadening ESW when paired with cathode-stable electrolytes. Applying an ultrathin Li_3_N coating on garnet-type LLZO reduces initial interfacial resistance by more than an order of magnitude, enabling stable operation for hundreds of hours in Li|Li symmetric cells and over 400 stable cycles in Li|LiFePO_4_ full-cells.^11,46^ Likewise, a Li_3_OCl coating on argyrodite suppresses electron penetration and lithium dendrite growth, delivering higher current density, longer cycle life, and superior rate capability compared to bare Li_6_PS_5_Cl in both symmetric and full cells.^47^ Similar improvements are observed when irreducible SE interlayers separate Li metal from halide or sulfide electrolytes, markedly extending cycle life.^48–51^

Notably, stable cycling does not necessarily indicate chemical compatibility. For example, sulfide-based SEs such as Li_6_PS_5_Cl rapidly form passivating layers at the Li metal^20^ interface, masking ongoing decomposition. Therefore, to assess true (electro)­chemical stability, it is important to combine electrochemical measurements with in situ or post-mortem structural, chemical, and morphological analyses, supported by atomic-scale simulations of interfacial reactivity at both anode and catholyte interfaces.^17,24,29^

Direct optical and spectroscopic measurements confirm that irreducible SEs remain inert to Li metal. Yu et al. observed no apparent color change in the Li|Li_2.5_N_0.5_S_0.5_|Li pellet after 12h of open-circuit aging (Figure 3d). Further exposing Li_2.5_N_0.5_S_0.5_ to molten Li resulted in neither detectable visual changes nor new phase formation, as confirmed by X-ray photoelectron spectroscopy.^17^ Similarly, post-mortem analysis of β-Li_3_N after cycling, using high-resolution scanning electron microscopy, X-ray absorption near edge structure, and scanning transmission X-ray microscopy, demonstrated no morphological or chemical degradation.^29^

To support experimental observations, AIMD simulations can provide atomic-scale insights into chemical stability and kinetic reactivity at the interfaces. For example, an Li_5_PS|Li interface remained stable during 100 ps of AIMD. Although this is very short compared to practical time scales, reducible electrolytes Li_7_P_3_S_11_ and Li_3_PS_4_ under the same conditions showed rapid SEI layer growth, driven by the dissolution of PS_4_^3–^ and P_2_S_7_^4–^ units.^30^ Similarly, AIMD simulations of Li_9_N_2_Cl_3_|Li interface showed no SEI formation or interfacial reactivity consistent with the anticipated electrochemical stability of Li_9_N_2_Cl_3_ toward Li metal.^52^

To assess chemical stability at interfaces between irreducible SEs (Li_3_N and Li_5_NCl_2_) and catholytes, Landgraf et al. employed a pseudobinary model^25^ (Figure 3e). The calculations showed significantly lower thermodynamic driving forces for decomposition in contact with garnet (LLZO), LIPON-type, and halide catholytes compared to sulfide or lithium aluminum titanium phosphate (LATP) SEs. Follow-up experiments confirmed stable Li_3_N|Li_3_YCl_6_ interface with no new phases and minimal interfacial resistance change during prolonged cycling.^29^

Combining irreducible SEs with cathode-stable electrolytes addresses oxidative stability challenges, but achieving true interface compatibility and long-term cycling performance necessitates integrated electrochemical characterization, advanced in situ or post-mortem analysis, and atomic-level simulations of interfacial reactions at both anode and catholyte interfaces.

^29^

Although systematic studies remain sparse, pairing irreducible SEs with cathode-stable electrolytes (e.g., garnet, LIPON, sulfide or halide SEs) clearly mitigates intrinsic oxidative stability limitations. Catholyte selection principles should be guided by (i) high oxidative stability to protect the cathode interface, (ii) fast Li-ion conductivity to mitigate transport bottlenecks, and (iii) thermodynamic and kinetic compatibility with irreducible SEs to prevent undesirable reactions. Halide catholytes such as Li_3_YCl_6_ and Li_3_InCl_6_ are particularly promising because of their high oxidative stability (>4.5 V) and relatively wide ESW, while sulfide catholytes like Li_6_PS_5_Cl offer excellent ionic conductivity (>10^–3^ S cm^–1^) and good processability. Further systematic research focusing on interfacial stability and long-term cycling remains crucial to facilitate the practical application of irreducible SEs.

Although the ionic conductivity and anode stability have advanced markedly, several aspects must still be addressed before irreducible SEs can be applied in practical systems. These include air stability and large-scale manufacturing of thin layers.

The anion framework of irreducible SEs typically comprises P^3–^, N^3–^, or S^2–^, which readily hydrolyze upon exposure to moisture, resulting in material decomposition, toxic gas evolution, and loss of ionic conductivity.^53–56^ Consequently, irreducible SE powders and films must be sufficiently stable under standard dry-room conditions to avoid the need for costly inert-atmosphere processing. Some nitride-based irreducible SEs remain stable under dry air, yet even trace humidity compromises their structure and conductivity.^57^ For example, vacancy-rich Li_9_N_2_Cl_3_ is stable at ∼3–5% relative humidity (RH) but degrades at ambient conditions (≈20% RH), forming Li_4_(OH)_3_Cl and NH_3_.^24^ Likewise, β-Li_3_N preserves its structure in dry air but rapidly acquires a self-passivating LiOH layer under modest moisture conditions.^29^ Although these cases suggest that today’s industrial dry rooms (<1% RH) are adequate for manufacturing irreducible SEs, a deeper understanding of moisture-induced degradation mechanisms and the design of intrinsically air-stable compounds remains essential.

Another critical factor for practical implementation is the gravimetric energy density which scales with both electrolyte’s density and its thickness. The irreducible SEs considered here (e.g., ρ_Li_3_N_ = 1.27 g cm^–3^; ρ_Li_2.5_N_0.5_S_0.5__ = 1.63 g cm^–3^; ρ_Li_9_N_2_Cl_3__ = 1.7 g cm^–3^)^24,26,29^ have densities comparable to, or even lower than common sulfide (ρ_Li_6_PS_5_Cl_ = 1.8 g cm^–3^)^58^ and halide (ρ_Li_6_YCl_3__ = 2.1 g cm^–3^)^59^ SEs. Thus, at a given thickness, these irreducible SEs do not inherently reduce energy density relative to common alternatives. Electrolyte thickness is equally important: thick electrolyte layers not only add mass, thereby lowering energy density, but also significantly increase cell impedance and overpotential.^60^ Irreducible SEs achieve conductivities of 0.1 mS cm^–1^, yet this is still 2 orders of magnitude below leading sulfide electrolytes.^26,27^ Therefore, minimizing SE layer thickness is imperative to maintain acceptable ohmic losses at practical C-rates. Cold-pressed laboratory pellets typically measure several hundred micrometers, far above the <30 μm target for high-energy, fast-charge batteries.^2,61^ Scalable roll-to-roll methods (e.g., tape casting, extrusion, and dry calendaring^62–64^ ) can achieve smaller thicknesses, but the binders and solvents involved in these processes may chemically react with irreducible SEs.^65,66^ Alternative additive-free methods (e.g., electrodeposition^46^ and atomic-layer deposition^67^) yield submicrometer films which renders them more prone to fail from short circuit due to lithium dendrite formation. Indeed, Li filament penetration depths of 2–10 μm have been reported for Li_3_N–LiF^49^ and Li_7_N_2_I^33^ interlayers after only ∼50 cycles. These findings underscore the need to suppress dendrite formation.

Thus, optimizing irreducible SEs for industrial applications demands simultaneous improvement in ionic conductivity, electrochemical stability, and mechanical resilience. Intrinsic anion properties, such as ionic radius, formal charge, electronegativity, polarizability, and chemical hardness (Figure 4a), offer useful guiding principles directly influencing diffusion and stability of irreducible SEs. For example, ionic radius and formal charge tune diffusion bottleneck size and Li^+^–anion Coulombic interactions.^27^ Larger, lower-charge anions (e.g., S^2–^) widen diffusion pathways, whereas smaller, highly charged anions (e.g., N^3–^) increase lithium concentration and introduce favorable structural disorder, accelerating ionic transport.^26,68^ Electronegativity sets the valence-band depth and therefore oxidative stability.^45^ Highly electronegative O^2–^ and F^–^ deepen the valence band and harden the lattice,^69^ whereas less electronegative ions (e.g., S^2–^) trade some oxidative robustness for faster Li^+^ mobility.^70^ The combined influence of polarizability and electronegativity is captured by ionization potential trends, which empirically rank binary irreducible SEs for oxidative stability (N^3–^ < P^3–^ < H^–^ ≪ S^2–^ < I^–^ < O^2–^ < Br^–^ < Cl^–^ ≪ F^–^).^39^ Recent work on Li_2.65_S_0.35_N_*x*_P_0.65‑x_ solid solutions illustrates how systematic and targeted anion substitution can exploit these trends to simultaneously enhance ionic conductivity and oxidative stability.^68^ Compared to reported irreducible SE families, this material achieves a standout ionic conductivity (>1 mS cm^–1^) coupled with an extended oxidation stability (1.15 V vs Li^+^/Li), enabling compatibility not only with Li but also within composite Si anodes. Although its chemo-mechanical compatibility during Si volume changes remains underexplored, this study provides a compelling example of how anion engineering within the antifluorite framework can balance ionic transport with stability. Moreover, polarizability and chemical hardness also govern lattice softness and air robustness,^70,71^ yet their influence on irreducible SEs remains largely unexplored. So far, only a handful of ternaries (and even fewer quaternary) compounds have been investigated, leaving an expansive compositional space unprobed.

Although irreducible SEs have mostly been explored with lithium metal anodes, they also hold significant promise for forming stable, SEI-free interfaces with alternative high-capacity anodes such as silicon, provided their electrochemical stability extends beyond 1 V vs Li/Li^+^ during cycling.

**Figure 4:**
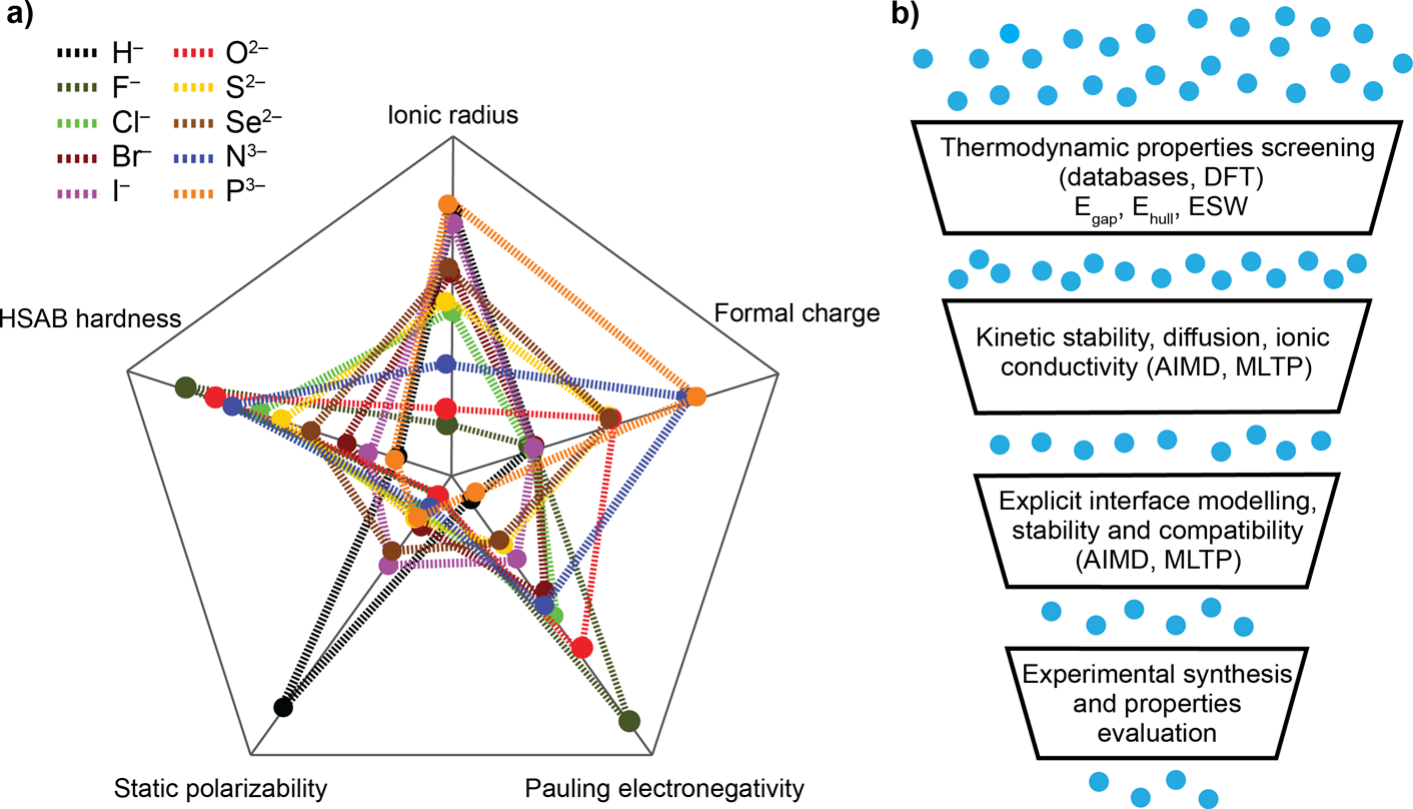
Screening and optimization strategies for irreducible SE design. (a) Key anion properties including ionic radius, formal charge, electronegativity, polarizability, and chemical hardness. (b) Schematic overview of computational screening workflow integrating DFT, MD, and machine learning (ML) techniques like ML trained potentials (MLTPs) to identify promising irreducible SE candidates.

Investigation of this wide compositional space calls for systematic computational screening (Figure 4b) coupled with targeted synthesis and properties characterization. Computational approaches, including DFT, AIMD, and ML, can rapidly evaluate thermodynamic stability, electronic structure, lattice softness, Li diffusion mechanisms, and bottleneck geometry, thereby flagging promising candidates prior to synthesis.^72–74^ High-throughput DFT screening of formation energies, band gaps, above-hull stabilities, ionic conductivities, and reaction energies has already guided the search for Li- and Na-ion conducting SE candidates^73^ and compatible coatings for halide SEs.^48^ For irreducible SEs, additional prescreening metrics, including bottleneck size, diversity of Li environments, and electrostatic interaction strength, can capture the effects of anion disorder. Experimental characterization, coupled with explicit interface modeling, is then required to assess chemical reactivity and Li-ion transport across heterointerfaces. To reach larger length and time scales, ML interatomic potentials enable nanosecond-to-microsecond simulations over hundreds of nanometers,^75^ which could be sufficient to probe early dendrite formation, optimize coating thickness, and track the initial evolution of SEI —all of which would be practically intractable by AIMD.^76,77^

Irreducible SEs in which all non-lithium elements are already in their lowest oxidation state exhibit intrinsic reduction stability. This property makes them attractive to pair with low-potential, high-capacity anodes such as Li metal and silicon. Their main weaknesses are (i) limited oxidative stability, which requires a cathode-stable protective layer; (ii) sensitivity to ambient moisture despite tolerance to dry-air conditions; and (iii) moderate ionic conductivity (commonly ≤ 0.2 mS cm^–1^), which requires thin-film processing. To resolve these concerns, we propose the following research directions:

1) Compositional engineering beyond traditional anions: The rational combination of anions with different ionic radii and electronegativity can simultaneously widen diffusion bottlenecks and deepen the valence band, thus improving both conductivity and oxidative stability. Integrating high-throughput DFT, AIMD, and ML potentials will accelerate screening of available vast chemical design space.
2) Investigating interfaces with cathode-stable electrolytes: Pairing irreducible SEs with a cathode-stable layer (e.g., halide or oxide SEs) demands systematic studies of interfacial thermodynamics, Li-ion transport, and redox chemistry both experimentally and through atomic-scale modeling.
3) Expanding application beyond Li metal anodes: While studies have primarily focused on Li metal anodes, irreducible SEs are promising to form decomposition-free interfaces with other high-capacity anodes (e.g., silicon).
4) Scalable thin-film processing: Atomic-layer deposition or electrodeposition can yield submicrometer irreducible SEs coatings required to maintain high-energy density and ionic conductivity.
